# The impact of maternal emotional warmth on adolescents’ internalizing problem behaviors: the roles of meaning in life and friendship conflict

**DOI:** 10.3389/fpsyg.2024.1478610

**Published:** 2024-11-28

**Authors:** Ludan Zhang, Ruijie Wang, Yaoyao Li, Liang Chen

**Affiliations:** ^1^Department of Development and Planning, Weifang University of Science and Technology, Weifang, China; ^2^School of Psychology, Shandong Normal University, Jinan, China

**Keywords:** maternal emotional warmth, internalizing problem behaviors, meaning in life, friendship conflict, adolescent

## Abstract

**Introduction:**

Numerous studies suggest that maternal emotional warmth is a critical protective factor against adolescents’ internalizing problem behaviors. However, the underlying mechanisms linking these variables remain unclear. Grounded in ecological systems theory, this study explores the impact mechanisms of social support (maternal emotional warmth), individual resource (meaning in life), and environmental factor (friendship conflict) on adolescents’ internalizing problem behaviors.

**Methods:**

A questionnaire survey of 474 adolescents in vocational school aged 15–24 assessed maternal emotional warmth, meaning in life, friendship conflict, and internalizing problem behaviors.

**Results:**

The results indicate that the meaning in life partially mediates the relationship between maternal emotional warmth and adolescents’ internalizing problem behaviors, with friendship conflict moderating the latter half of this mediation pathway.

**Discussion:**

The findings suggest that adolescents, during their social adaptation process, activate different protective factors depending on the environmental relational context. Specifically, high friendship conflict limits the direct protective role of maternal emotional warmth, whereas a meaning in life becomes a significant protective factor, exerting its effect through mediation. Conversely, when friendship conflict is low, maternal emotional warmth directly serves as a protective factor.

## Introduction

Adolescents’ internalizing problem behaviors refer to unpleasant or negative emotional experiences, such as depression, anxiety, and withdrawal (Achenbach, 1991). Adolescence, a transitional period in life, is characterized by physiological, cognitive, and emotional changes (Cameron, 2004). Emotional problems are prevalent among adolescents and significantly impact their daily lives (Van Oort et al., 2009; Salk et al., 2016). Compared with externalizing problem behaviors, internalizing problem behaviors are more covert and often overlooked by parents and teachers, leading to insufficient evaluation and intervention. A large-scale study of European adolescents (11–16 years) reported that nearly one-fifth of Dutch adolescents experienced internalizing problem behaviors (Luijten et al., 2021), whereas a study of Chinese high school students revealed that over half (55.3%) of these individuals exhibited internalizing emotional symptoms (depression, anxiety, or compulsiveness), with detection rates of 34.9, 41.9, and 20.6% for depression, anxiety, and compulsiveness, respectively (Fang et al., 2019). Reviews have shown increasing rates of emotional symptoms in Chinese students in recent years (Yu et al., 2016). This directly endangers adolescent mental health; unevenly affects learning, relationships, and hobbies; and can lead to suicidality, causing heavy societal losses and impacts.

In the developmental process of adolescents, mothers play a crucial role (Curcio et al., 2018; Ken and Guan, 2023). Compared to fathers, mothers usually spend more time with adolescents, and are more involved in family life, assuming greater caregiving and educational responsibilities in daily life. This prolonged interaction provides a foundation for establishing a deep emotional connection between mothers and adolescents, making maternal emotional support more enduring and stable. Furthermore, compared to fathers, mothers typically express emotions more delicately (Keskin and Branje, 2022; Wang et al., 2018; Del Barrio et al., 2016), and this nuanced emotional support has a more significant impact on adolescents’ internalizing problem behaviors. Therefore, this study focuses on maternal emotional warmth as the research variable to examine its predictive effect on adolescents’ internalizing problem behaviors.

## Maternal emotional warmth and adolescents’ internalizing problem behaviors

Maternal emotional warmth is a protective factor against the occurrence and development of adolescents’ internalizing problem behaviors (Rothenberg et al., 2020). Life course theory emphasizes the importance of the family in the early stages of individual life. Maternal emotional warmth directly influences adolescents’ psychological development, with effects extending into adulthood. Studies indicate that parent–child interactions characterized by warmth and care are crucial for adolescents’ psychological adjustment during challenging periods (Gniewosz et al., 2023). Higher maternal emotional warmth scores are correlated with fewer internalizing and externalizing problem behaviors in adolescents (Alegre et al., 2013; Keijser et al., 2020). High scores for maternal emotional warmth are associated with fewer types of psychological health issues (depression, anxiety, and compulsiveness) (Fang et al., 2019). A study investigating the relationships among maternal emotional warmth, adolescent brain function, anxiety, and depressive symptoms revealed that maternal emotional warmth helps prevent future anxiety and depression among adolescents with a history of anxiety (Butterfield et al., 2021). Hence, this study posits that maternal emotional warmth plays a significant role in preventing adolescents’ internalizing problem behaviors.

## The mediating role of meaning in life

The meaning in life refers to an individual’s perception of their life’s meaning and value, and their awareness of clear goals and missions in life (Steger and Kashdan, 2013). It is a vital concept in positive psychology. Frankl first introduced the meaning in life from a personal psychological perspective, asserting that the primary human motivation is the endeavor to discover or realize meaning in life, with the lack of such meaning being a major cause of psychological problems (Frankl, 1963). Individuals with a low meaning in life often struggle to find reasons for their existence, tend to give up in the face of stress, experience helplessness, and exhibit more internalizing problem behaviors such as depression, anxiety, and withdrawal, along with greater suicidal thoughts (Steger, 2009). Schulenberg’s (2004) research revealed significant negative correlations between symptoms such as depression, anxiety, substance abuse, and the meaning in life. Steger (2009) study yielded similar results, showing strong negative correlations between a meaning in life and negative emotions such as sadness and depression. Previous studies have demonstrated that meaning in life significantly predictes internalizing problem behaviors (Bernasco et al., 2022). Clinical trials have also shown that meaning in life has relatively positive therapeutic effects, alleviating depressive symptoms in clinically depressed individuals (Mascaro and Rosen, 2008).

According to affect system theory (Mischel and Shoda, 1995), maternal emotional warmth promotes the development of a healthy emotional system in adolescents, enhancing their positive emotional experiences and thereby increasing their meaning in life while reducing their internalizing problem behaviors. Empirical studies indicate that meaning in life mediates the relationship between family factors and psychological health (Shen and Zhang, 2020). For example, research has shown that meaning in life mediates the relationship between family intimacy and positive psychological health indicators (e.g., subjective well-being) (Shen and Zhang, 2020), as well as between negative childhood experiences and negative psychological health indicators (e.g., depression) (Yu et al., 2020). Hence, these studies suggest that meaning in life may mediate the relationship between maternal emotional warmth and adolescents’ internalizing problem behaviors.

## The moderating role of friendship conflict

Ecological systems theory posits that individual development is influenced by interactions among multiple systems (Bronfenbrenner, 2000). Family and friends are the most crucial microsystems for adolescents (Zhou et al., 2023). Friendship plays a vital role in most adolescents’ social lives (Burk and Laursen, 2005). Friendship quality is a key indicator of adolescents’ psychological adjustment, with friendship conflict being an inevitable aspect of social relations and a common social stress. Most adolescents experience this negative stimulus (Chung et al., 2011). Friendship conflict typically reflect significant relationship characteristics (Laursen et al., 1996). Friendship is crucial in shaping adolescents’ behaviors and influencing self-well-being (Alsarrani et al., 2022). Research shows that adolescents with internalizing symptoms, such as depression and anxiety, often face higher rates of friendship instability (Guimond et al., 2019). Additionally, studies indicate that friendship quality moderates the relationship between external stressors (e.g., peer victimization) and internalizing problem behaviors, high-quality friendship characterized by support can buffer against the negative effects of victimization on internalizing symptoms; however, conflict within friendship can weaken this protective effect (Siennick and Picon, 2020). Therefore, these studies suggest that friendship conflict moderates the relationship between maternal emotional warmth and internalizing problem behaviors.

**Figure 1:**
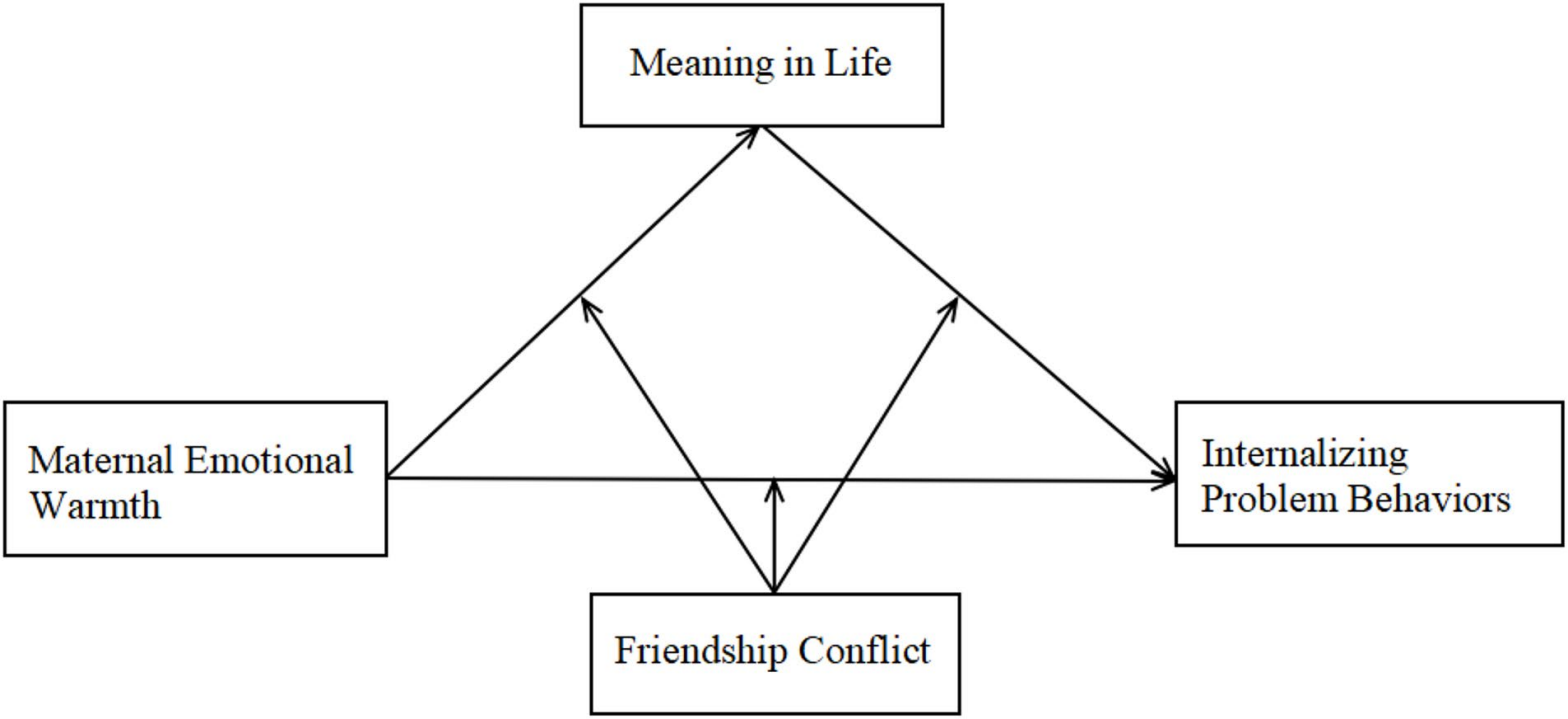
Conceptual model.

Previous research has partially explored the relationship between maternal emotional warmth and adolescents’ internalizing problem behaviors. However, few studies have focused on the mechanisms of this relationship, especially within the framework of ecological systems theory, examining the combined effects of family and friends. Moreover, past research has primarily focused on positive dimensions of friendship quality, such as intimacy, and their predictive effect on adolescents’ problem behaviors (Shulman et al., 1997; Van Zantvliet et al., 2020), neglecting the effects of negative dimensions, such as conflict. According to stress and coping Theory (Lazarus and Folkman, 1984), individual mental health and adaptation are determined by the interaction of multiple factors, including social support, individual resources, and environmental factors. These factors interact in complex ways, with individual mental health and social adaptation depending on the interplay between personal characteristics or abilities and stress events and environmental factors. On the basis, the present study aims to construct a moderated mediation model, simultaneously examining the relationships between maternal emotional warmth, meaning in life, friendship conflict, and adolescents’ internalizing problem behaviors. Specifically, this study investigates the mediating (meaning in life) and moderating (friendship conflict) mechanisms through which maternal emotional warmth predicts adolescents’ internalizing problem behaviors. The theoretical hypotheses of this study are as follows:

*H1*: Maternal emotional warmth negatively predicts adolescents’ internalizing problem behaviors.
*H2*: Meaning in life mediates the relationship between maternal emotional warmth and adolescents’ internalizing problem behaviors.
*H3*: Friendship conflict moderates the mediating role of meaning in life between maternal emotional warmth and adolescents’ internalizing problem behaviors. The theoretical model is shown in Figure 1.

## Materials and methods

### Participants

Use the formula *N* = Z^2^ × [*P* × (1-*P*)]/*E*^2^ for sample size estimation, where *N* is the required sample size, *Z* is the statistical measure, *E* is the error value, and *P* is the probability value. In this study, *Z* = 1.96 (confidence level selected as 95%), *E* = 0.05, and based on previous research, the detection rate of internalizing problematic behaviors was 20% (Yu and Huang, 2023). Therefore, the minimum required sample size is 246 people.

A cluster sampling method was used to measure adolescents from a vocational school in Shandong Province, China. After the purpose and procedure of this study are explained to the school and the school’s consent to the university is obtained, the graduate students who have undergone unified training will be the main examiners for the test. Before the start of the study, we obtained informed consent from the students’ parents and their own informed consent and informed them that they could withdraw from the study at any time. The test was conducted on a class basis, and the questionnaires were collected uniformly. Finally, the participants were given a gift worth 5 RMB (USD 0.73).

A total of 474 questionnaires were collected, of which 429 adolescents (*M*_age_ = 17.21, *SD* = 1.00) were valid and were included in the analyses. Table 1 shows the demographic characteristics of the participants.

**Table 1:** Sample characteristics (*N* = 429).

Characteristics	*N*	%	
Sex	Male	130	30.30
	Female	299	69.70
Grade	Freshman	141	32.87
	Sophomore	288	67.13
Father’s education	Junior high school or less	290	69.71
	Senior high school	97	23.32
	College or more	29	6.97
Mother’s education	Junior high school or less	329	78.71
	Senior high school	82	19.62
	College or more	7	1.67
Father’s occupation	Farmers or unemployed	121	30.56
	Semi professionals	182	45.96
	Professionals	93	23.48
Mother’s occupation	Farmers or unemployed	192	48.73
	Semi professionals	102	25.89
	Professionals	100	25.38
Family income (monthly)	<3,000 RMB	153	37.59
	3,000–6,000 RMB	163	40.05
	>6,000 RMB	91	22.36

Due to missing data, the total number of people in certain categories accounts for less than 100%.

### Variables and measures

#### Maternal emotional warmth

Maternal emotional warmth was measured using the Egna Minnen av. Barndoms Uppfostran (EMBU) scale which was originally developed by Perris et al. (1980) and revised by Yue et al. (1993). The full scale tests the father’s parenting style with 58 items divided across six dimensions and the mother’s parenting style with 57 items divided across five dimensions. For the purpose of this study, we only used the “emotional-warmth” dimension of the mother’s subscale. It includes 19 items (e.g., “If I am facing a difficult task, I can feel the support from my mother”), which participants respond to on a 4-point Likert scale (1 = never; 4 = always). The scale score was the average across the 19 questions, and the higher the score, the greater the level of emotional warmth perceived by adolescents from their mothers. The Cronbach’s alpha was 0.93 in this study.

#### Meaning in life

The meaning in life of adolescents was measured via the Meaning in Life Questionnaire (MLQ) initially developed by Steger et al. (2006), which is reliable among Chinese adolescents (Wang, 2013). The scale includes 10 items (e.g., “I have a clear sense of purpose in my life”) and participants respond to each item via a 5-point Likert scale (1 = very inconsistent; 5 = very consistent). The scale score was the average across the 10 questions, and the higher the score, the higher the meaning in life for adolescents. The Cronbach’s alpha was 0.82 in this study.

#### Internalizing problem behaviors

Internalizing Problem Behaviors were measured via the Chinese version of the Youth Self-Report scale (YSR, Achenbach and Rescorla, 2001) which has been proven to be effective and reliable in the context of Chinese culture (Wang et al., 2013). The scale includes 54 items, including the externalizing problem behaviors subscale which includes aggression and disciplinary violations dimensions and the internalizing problem behaviors subscale which includes anxiety/depression and withdrawal/depression dimensions. For research purposes, we only used the internalizing problem behaviors subscale, where anxiety/depression included 13 items (e.g., “I feel useless and inferior to others”), and the withdrawal/depression dimension included 8 items (e.g., “I like being alone and do not want to be with others”), for a total of 21 items. The participants responded to each item via a 3-point Likert scale (1 = not at all or never, 3 = very much or often). The scale score was the average across the 21 questions, and the higher the score, the more serious the internalizing problem behaviors of adolescents. The Cronbach’s alpha was 0.88 in this study.

#### Friendship conflict

Adolescents’ friendship conflict was measured via the Network of Relationships Inventory (NRI, Furman and Buhrmester, 1985) which was reliable in the context of Chinese culture (Tian et al., 2012). The scale involves the relationship between adolescents and their most important same-sex friends, including five dimensions: companionships, instrumental help, annoyance and conflict, intimacy, and affection, for a total of 15 items. Based on the research purpose, this study used only the dimensions of annoyance and conflict for measurement, which consists of 3 items (e.g., “How much do you and this person disagree and quarrel”). The participants responded to each item via a 5-point Likert scale (1 = few or never, 5 = very much). The scale score was the average across the 3 questions, and the higher the score, the more arguments and conflicts teenagers have with their peers. The Cronbach’s alpha was 0.82 in this study.

### Data analysis

This study used SPSS 26.0 and PROCESS 4.1 to analyze the descriptive statistics, correlations, mediating effects, and moderating effects tests on the collected data. First, Pearson correlation analysis was used to analyze the correlations between various variables, and then the PROCESS macro (Model 59) (Hayes, 2013) was used to test the mediating and moderating effects. Among them, gender was the controlling variable, maternal emotional warmth was the independent variable, meaning in life was the mediating variable, friendship conflict was the moderating variable, and internalizing problem behaviors were the dependent variable. Finally, simple slope and Johnson–Neyman method analyses are performed on the significant interaction terms.

## Results

### Common method deviation analysis

Harman’s single-factor test was used to identify common method deviation. Principal component analysis revealed 14 factors with eigenvalues greater than 1. The first factor explained a variation of 18.16%, which was less than the critical standard of 40% (Deng et al., 2018). This indicated that there was no serious problem of common method bias in the data and that subsequent analysis could be conducted.

### Descriptive statistics

The Pearson correlation method was used to analyse gender, maternal emotional warmth, meaning in life, friendship conflict, and internalizing problem behaviors. The results indicated (see Table 2) that maternal emotional warmth was significantly and positively associated with meaning in life (*r* = 0.256, *p* < 0.001), while was negatively associated with internalizing problem behaviors (*r* = −0.157, *p* < 0.001). And meaning in life was significantly and negatively associated with internalizing problem behaviors (*r* = −0.159, *p* < 0.001). Researches have shown that gender could predict internalizing problem behaviors (Balaban and Bilici, 2022; Sun et al., 2023), so subsequent analysis used gender as a control variable.

**Table 2:** Descriptive statistics and correlation analysis (*N* = 429).

	*M* ± *SD*	1	2	3
1. Maternal emotional warmth	2.563 ± 0.573	1		
2. Meaning in life	3.561 ± 0.581	0.256^***^	1	
3. Friendship conflict	1.815 ± 0.699	−0.044	0.005	1
4.Internalizing problem behaviors	1.557 ± 0.342	−0.157^***^	−0.159^***^	0.098

^***^p < 0.001.

### Mediation analysis

Maternal emotional warmth significantly negatively predicted their internalizing problem behaviors (*β* = −0.147, *p* = 0.003). After meaning in life was added, maternal emotional warmth still predicted their internalizing problem behaviors (*β* = −0.113, *p* = 0.026), which indicated that the meaning of life partially mediated between maternal emotional warmth and internalizing problem behaviors. Then we generated 5,000 bootstrapping samples from the original data set (*N* = 429) via random sampling to test the mediating effect. The results (see Table 3) indicated that meaning in life played a mediating role between maternal emotional warmth and internalizing problem behaviors [Index = −0.034, SE = 0.017, 95% CI = (−0.072, −0.004)]. Thus, meaning in life mediated the relationship between maternal emotional warmth and internalizing problem behaviors, with an effect size (the ratio of indirect to total effects) of 23.28%, indicating that with respect to the impact of maternal emotional warmth on internalizing problem behaviors, 23.28% passed through the mediating pathway of meaning in life.

**Table 3:** Testing the mediating effect of meaning in life on mother emotional warmth and internalizing problem behaviors.

Path	Effect	SE	95% CI		Effect Size
			Lower	Upper	
Total effect	−0.089	0.030	−0.147	−0.030	
Direct effect	−0.068	0.031	−0.128	−0.008	76.72%
Indirect effect	−0.021	0.011	−0.044	−0.003	23.28%

### Moderation analysis

**Figure 2:**
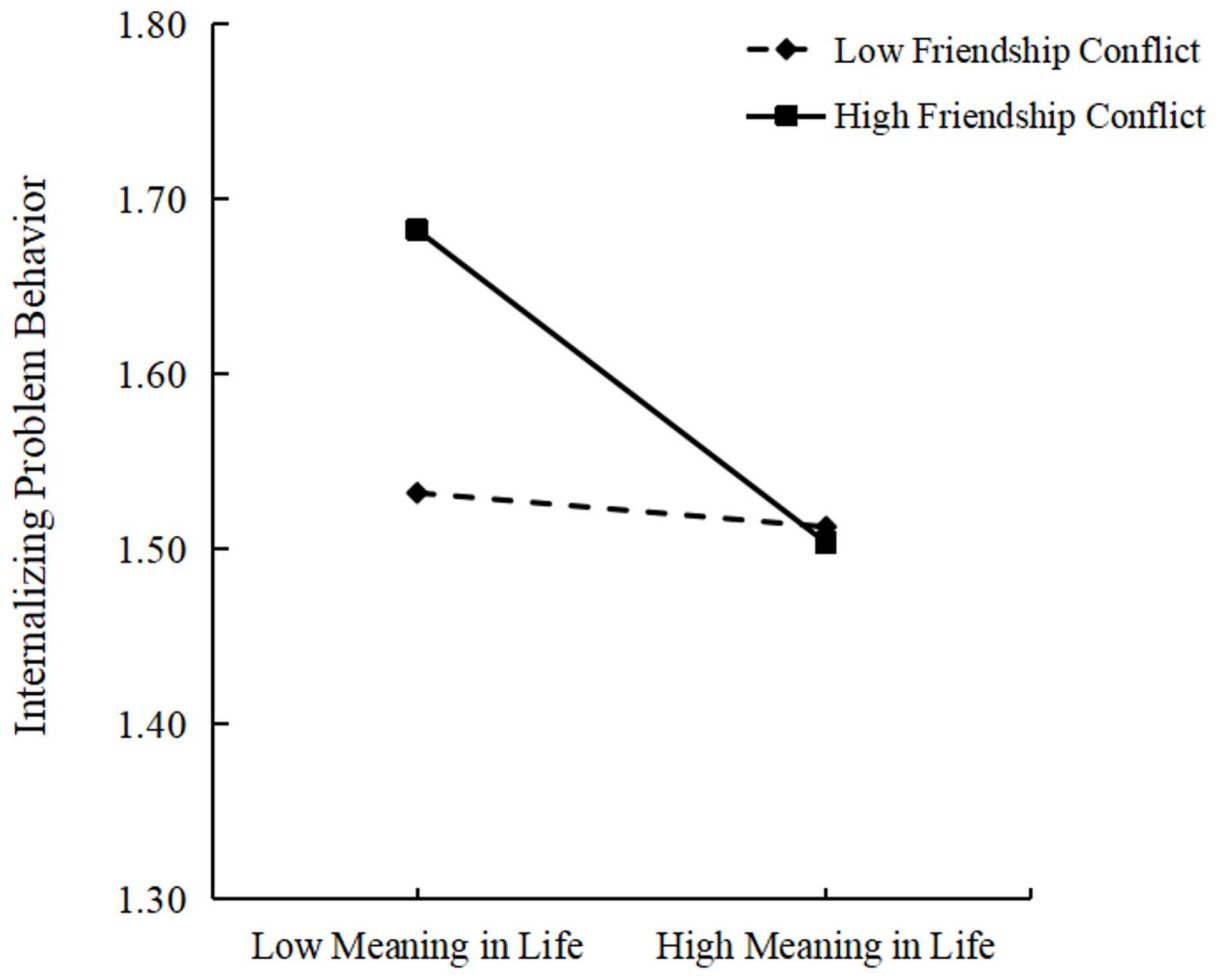
Interaction between meaning in life and in the prediction of internalizing problem behavior. Low conflict: 1 *SD* below mean score of friendship conflict; High conflict: 1 *SD* above mean score of friendship conflict; Low meaning in life: 1 *SD* below mean score; High meaning in life: 1 *SD* above mean score.

As shown in Table 4, friendship conflict moderated the relationship between maternal emotional warmth and internalizing problem behaviors. After friendship conflict was incorporated into the model, the interaction between maternal emotional warmth and friendship conflict did not have a significant predictive effect on meaning in life. The interaction between meaning in life and friendship conflict had a significant predictive effect on internalizing problem behaviors [*β* = −0.097, SE = 0.044, 95% CI = (−0.183, −0.011)], indicating that friendship conflict could moderate the relationship between meaning in life and internalizing problem behaviors. The results of *post hoc* simple slope analyses (see Figure 2) indicated that among those with low (*M* - 1*SD*) friendship conflict, there was no significant predictive effect on internalizing problem behaviors in terms of meaning in life (*B*_simple_ = −0.017, *t* = −0.403, *p* = 0.688). Among those with high (*M* + 1*SD*) friendship conflict, there was a significantly negative predictive effect on internalizing problem behaviors in terms of meaning in life (*B*_simple_ = −0.153, *t* = −3.407, *p* < 0.001). The results indicated that compared to low-level friendship conflicts, the meaning in life of adolescents had a stronger predictive effect on internalizing problem behaviors during high-level friendship conflict.

**Table 4:** Testing the moderating effect of friendship conflict on the relationship between mother emotional warmth and internalizing problem behaviors via meaning in life.

Variable	Outcome variable IBP			Outcome variable ML			Outcome variable IBP		
	*β*	*t*	95% CI	*β*	*t*	95% CI	*β*	*t*	95% CI
Gender	0.072	1.457	[−0.021, 0.128]	0.048	0.752	[−0.078, 0.175]	0.065	1.712	[−0.010, 0.141]
MEW	−0.147	−2.986^**^	[−0.149, −0.025]	0.291	5.700^***^	[0.190, 0.391]	−0.047	−1.491	[−0.109, 0.015]
FC				0.019	0.470	[−0.062, 0.100]	0.051	2.070^*^	[0.003, 0.099]
MEW×FC				0.015	0.211	[−0.123, 0.153]	0.034	0.794	[−0.050, 0.118]
ML							−0.085	−2.798^**^	[−0.144, −0.025]
ML × FC							−0.097	−2.227^*^	[−0.183, −0.011]
*R* ^2^	0.030			0.077			0.062		
*F*	6.302^**^			8.126^***^			4.244^***^		

MEW, mother emotional warmth; ML, meaning in life; FC, friendship conflict; IPB, internalizing problem behaviors.

^*^p < 0.050; ^**^p < 0.010; ^***^p < 0.001.

**Figure 3:**
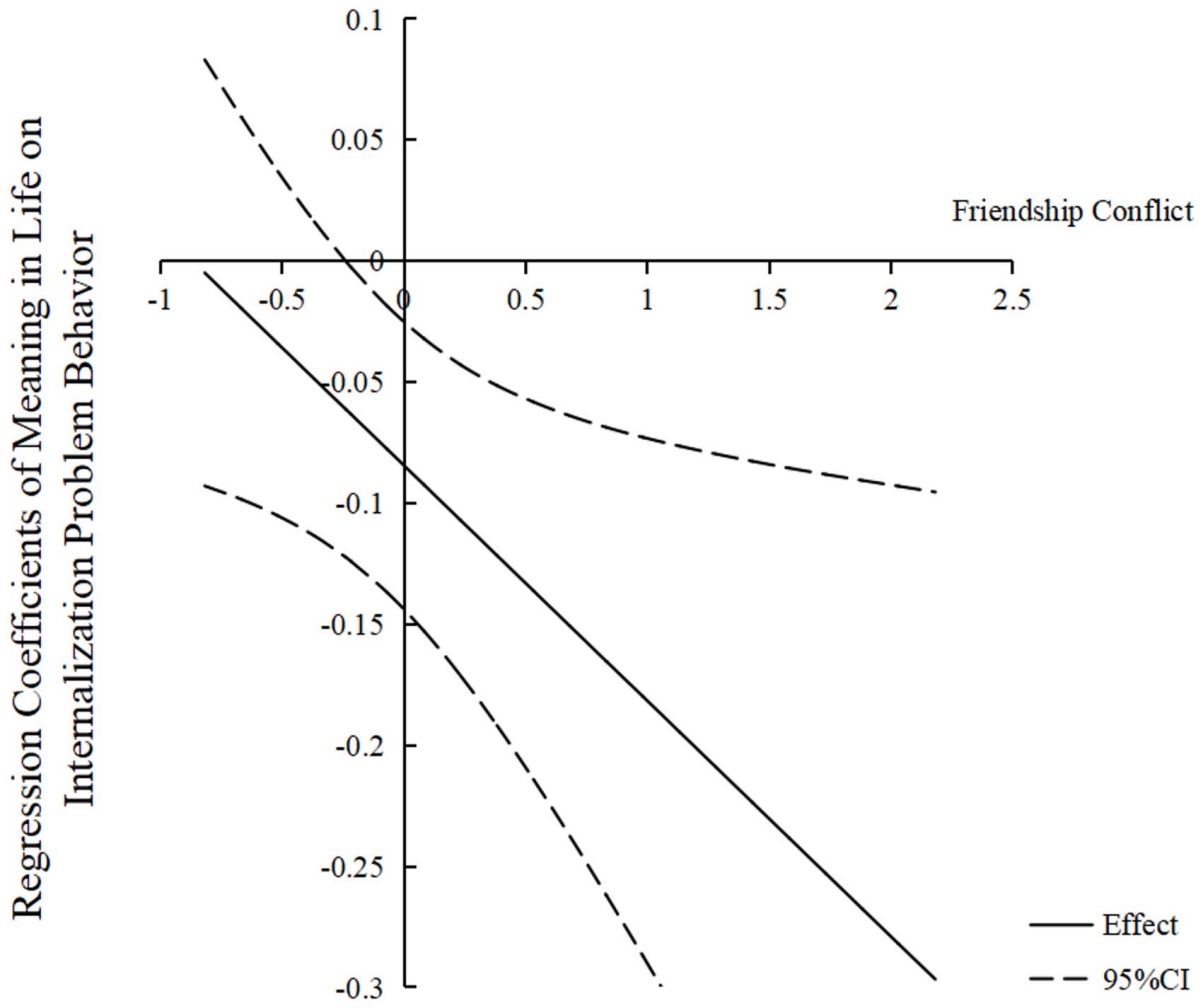
The moderating effect of friendship conflict on the relationship between meaning in life and internalizing problem behavior. The numbers on the *X*-axis represent the standardized values of friendship conflict. The numbers on the *Y*-axis represent the regression coefficient values of meaning in life on internalizing problem behavior. The middle line is the point estimation. The two curves up and down are the values of the 95% confidence intervals.

Moreover, the Johnson–Neyman method was used to test the significance of statistical moderation at the transition point. The results (see Figure 3) indicated that in the range of −0.818 to −0.240 levels of friendship conflict, the regression coefficient values of meaning in life on internalizing problem behaviors were not significant. In the range of −0.240 to 2.182 levels of friendship conflict, the regression coefficient values of meaning in life on internalizing problem behaviors were significant according to simple slope tests and all the values were less than zero. These results indicated that in the range of −0.818 to −0.240 levels of friendship conflict, the predictive effect of meaning in life on internalizing problem behaviors was not significant. Conversely, in the range of −0.240 to 2.182 levels of friendship conflict, meaning in life had a significant negative predictive effect on internalizing problem behaviors, and the effect appeared uptrend along with the increase of friendship conflict.

In addition, as shown in Table 5, when the level of friendship conflict was low, the mediating effect of meaning in life on the relationship between maternal emotional warmth and internalizing problem behaviors was not significant [Index = −0.005, SE = 0.015, 95% CI = (−0.037, 0.025)]. When the level of friendship conflict was high, the mediating effect of meaning in life on the relationship between maternal emotional warmth and internalizing problem behaviors was significant [Index = −0.046, SE = 0.019, 95% CI = (−0.087, −0.015)]. This finding indicated that when adolescents had a high level of friendship conflict, their maternal emotional warmth could indirectly predict their internalizing problem behaviors by influencing their meaning in life.

**Table 5:** Testing the mediating effect at different levels of friendship conflict.

Friendship conflict	Effect	SE	95% CI	
			Lower	Upper
*M* - 1*SD*	−0.005	0.015	−0.037	0.024
*M* + 1*SD*	−0.046	0.019	−0.090	−0.016

## Discussion

This study primarily explored the predictive effects of maternal emotional warmth, meaning in life, and friendship conflict on adolescents’ internalizing problem behaviors. The findings indicate that meaning in life mediates the effect of maternal emotional warmth on internalizing problem behaviors, whereas friendship conflict moderates this mediating effect.

### The relationship between maternal emotional warmth and adolescents’ internalizing problem behaviors

Consistent with our hypothesis, maternal emotional warmth and understanding significantly negatively predict adolescents’ internalizing problem behaviors. Adolescence is known to be a risk period for depression and anxiety (Hofmann et al., 2012; Yap et al., 2014). According to attachment theory (Cassidy and Shaver, 1999), maternal emotional warmth can help adolescents establish secure attachment relationships, promote healthy emotional development and reduce internalizing problem behaviors. Previous research also supports this conclusion, for example, warm, supportive, and responsive parenting behaviors are related to better adjustment and overall well-being in young adulthood (Zimmermann et al., 2008; Liu et al., 2024; Martínez-Castilla et al., 2023). Parental warmth has been found to protect against future anxiety and depressive symptoms in adolescents with a history of anxiety (Butterfield et al., 2021). Maternal emotional warmth, involvement, and autonomy support buffer youth from depression (Sander and McCarty, 2005). Moreover, a positive parenting style that includes warmth, structure, and autonomy support is associated with fewer depressive symptoms in adolescents (Keijser et al., 2020). Therefore, understanding the impact mechanisms of maternal emotional warmth on adolescents’ internalizing problem behaviors can aid in the intervention and prevention of internalizing issues such as depression and anxiety in adolescents.

### The mediating role of meaning in life

The results indicate that the meaning in life partially mediates the effect of maternal emotional warmth on adolescents’ internalizing problem behaviors. In other words, maternal emotional warmth can directly negatively predict internalizing problem behaviors and can also predict them through the meaning in life. Consistent with previous research findings, the more support individuals receive from family, school, and society, the greater meaning in life they have (Chen and Li, 2015). Parental rearing styles are closely related to the meaning in life (Callan, 1987). Parents who adopt an emotionally warm parenting style provide encouragement and support when adolescents face significant choices and difficulties, which serves as a powerful force driving them to explore their life’s value and meaning. The level of meaning in life influences an individual’s mental health, as it is a beneficial factor in maintaining psychological health. Individuals with greater meaning in life can effectively reduce negative emotions such as anxiety and depression (Yek et al., 2017). This study also supports the affect system theory, which posits that emotions are not only reactions but also integral components of the motivational system (Vandercammen et al., 2014). Maternal emotional warmth can evoke positive emotions in adolescents, increasing their intrinsic motivation to pursue meaningful goals and lifestyles. According to self-determination theory (Ryan and Deci, 2000) and social support theory (Vaux, 1989), maternal emotional warmth can fulfill adolescents’ needs for relatedness and social support, thus fostering their meaning in life. The enhancement of the meaning in life further supports adolescents’ mental health, reducing the occurrence of internalizing problem behaviors. Therefore, in educational practice, improving maternal rearing styles can motivate adolescents’ meaning in life, thereby reducing internalizing problem behaviors.

### The moderating role of friendship conflict

Friendship conflict encompasses various forms of negative interactions, such as arguments, disputes, and emotional distress resulting from differing opinions or expectations. It plays a significant moderating role in the mediating effects of maternal rejection, meaning in life, and adolescents’ internalizing problem behaviors. The findings reveal that friendship conflict significantly moderates the mediating effect of meaning in life. The moderating effect indicates that greater friendship conflict strengthens the predictive effect of a meaning in life on internalizing problem behaviors. Specifically, when friendship conflict is high, the negative predictive effect of meaning in life on internalizing problem behaviors is strongest. Conversely, when friendship conflict is low, the negative predictive effect weakens or disappears. This result may be explained by resource-based theory (Coff, 1997), which suggests that under high levels of friendship conflict, individuals face more emotional stress and challenges, making the meaning in life a crucial coping resource. The meaning in life can provide psychological support and stability, helping individuals better manage the negative impacts of conflict, thereby enhancing its negative predictive effect on internalizing problem behaviors. In contrast, when friendship conflict is low, the stress from conflict is minimal, rendering the meaning in life less critical; thus, its predictive effect weakens or disappears.

Previous research has shown that friendship quality enhances the protective effects of meaning in life (Alsarrani et al., 2022). However, this study revealed that as friendship conflict increases (indicating lower friendship quality), the protective effect of the meaning in life strengthens. This phenomenon may be attributed to adolescents’ differential perceptions of the positive and negative dimensions of friendship. According to prospect theory (Kahneman and Tversky, 1979), people’s perceptions of potential losses and gains are asymmetrical; specifically, people are more sensitive to losses from negative stimuli than to gains from positive stimuli. Therefore, under increased friendship conflict, negative stimuli become more prominent, and adolescents’ perceptions of conflict and stress in friendships are heightened. In such situations, the meaning in life, as an internal resource, is more strongly activated to help adolescents cope with these negative contexts, thereby enhancing its protective effect. When friendship quality is high and conflict is minimal, adolescents already receive substantial emotional support and positive experiences. Although the protective effect of the meaning in life still exists, it is less pronounced than in high-conflict situations. Thus, under increased friendship conflict, the negative predictive effect of the meaning in life is stronger.

Moreover, the moderated mediation effect test indicates that when individuals are in high friendship conflict environments, maternal emotional warmth is associated with enhanced meaning in life, which in turn is related to a reduction in internalizing problem behaviors. Conversely, in low friendship conflict environments, this mediating effect is absent, and maternal emotional warmth is directly associated with internalizing problem behaviors. This finding aligns with the compensatory hypothesis of protective factors, which posits that different protective factors may have competitive effects on the basis of environmental needs. When the effect of one protective factor is limited by the environment, another protective factor is activated to compensate (Masten et al., 1990). In high friendship conflict environments, meaning in life significantly mediates the relationship between maternal warmth and internalizing problem behaviors, with maternal warmth exerting an indirect effect on adolescents’ internalizing problems behaviors through meaning in life. Conversely, in low friendship conflict environments, the mediating role of meaning in life is not significant. This reflects adolescents’ social adaptation, demonstrating that individuals activate different protective factors in response to varying environmental relational contexts.

This study reflects the complex role of maternal emotional warmth in the developmental processes of adolescents, highlighting the influence of familial, individual, and peer factors on internalizing problem behaviors. Although the observed effect sizes in this research are relatively small, this does not necessarily diminish the practical significance of the findings. The size of the effect is often related to the research context, sample characteristics, and the complexity of the variables involved. Future studies could further explore the relationship between maternal emotional warmth and adolescents’ internalizing problem behaviors by increasing sample sizes, selecting different measurement tools, or introducing additional variables to validate and expand the research conclusions.

## Implications

This study, which is based on ecological systems theory and stress and coping theory, examines the effects of social support (maternal emotional warmth), individual resources (meaning in life), and environmental factors (friendship conflict) on adolescents’ internalizing problem behaviors. This study reveals significant factors and mechanisms influencing adolescent social adaptation, with key research implications:

First, the meaning in life is an important psychological resource for improving adolescents’ internalizing problem behaviors. The implementation of life education courses can help students appropriately understand and manage negative stimuli in life. Life education offers a novel perspective for adolescent mental health education, providing deeper insights and broader implications than conventional mental health education does, particularly when adolescents face high friendship conflict. Timely provision of life education and activities that help them find intrinsic motivation is crucial.

Second, the moderating effect of friendship conflict expands the theoretical understanding of family influences on adolescents’ internalizing problem behaviors, clarifying the complexity of interactions among microsystems (family, individual, friends) in adolescents’ development. Identifying the risk and protective factors for internalizing problem behaviors enables better intervention strategies, enhancing adolescents’ overall social adaptation capabilities.

### Limitations and future directions

This study has certain limitations. First, it primarily employs cross-sectional data and lacks accuracy in causal inferences. Future research should use longitudinal tracking to improve scientific rigor. Second, the sample size is relatively small and relies on self-reported data. Future studies should incorporate multisource data (e.g., reports from parents, teachers, peers) and increase sample sizes to increase reliability. Third, cultural differences might affect the results; the predictive effects of maternal emotional warmth, a meaning in life, and friendship conflict on adolescents’ internalizing problem behaviors might vary across cultural contexts. Cross-cultural studies can help verify and extend the findings.

## Data Availability

The raw data supporting the conclusions of this article will be made available by the authors, without undue reservation.
